# Fast quantification of gut bacterial species in cocultures using flow cytometry and supervised classification

**DOI:** 10.1038/s43705-022-00123-6

**Published:** 2022-04-25

**Authors:** Charlotte C. van de Velde, Clémence Joseph, Anaïs Biclot, Geert R. B. Huys, Vitor B. Pinheiro, Kristel Bernaerts, Jeroen Raes, Karoline Faust

**Affiliations:** 1 KU Leuven, Department of Microbiology, Immunology and Transplantation, Rega Institute for Medical Research, Laboratory of Molecular Bacteriology, B-3000 Leuven, Belgium; 2 VIB-KU Leuven, Center for Microbiology, B-3000 Leuven, Belgium; 3 KU Leuven, Department of Pharmaceutical and Pharmacological Sciences, Rega Institute for Medical Research, Medicinal Chemistry, B-3000 Leuven, Belgium; 4 KU Leuven, Department of Chemical Engineering, Chemical and Biochemical Reactor Engineering and Safety (CREaS), B-3001 Leuven, Belgium

**Keywords:** Microbial ecology, Microbiology

## Abstract

A bottleneck for microbial community experiments with many samples and/or replicates is the fast quantification of individual taxon abundances, which is commonly achieved through sequencing marker genes such as the 16S rRNA gene. Here, we propose a new approach for high-throughput and high-quality enumeration of human gut bacteria in a defined community, combining flow cytometry and supervised classification to identify and quantify species mixed in silico and in defined communities in vitro. We identified species in a 5-species in silico community with an F1 score of 71%. In addition, we demonstrate in vitro that our method performs equally well or better than 16S rRNA gene sequencing in two-species cocultures and agrees with 16S rRNA gene sequencing data on the most abundant species in a four-species community. We found that shape and size differences alone are insufficient to distinguish species, and that it is thus necessary to exploit the multivariate nature of flow cytometry data. Finally, we observed that variability of flow cytometry data across replicates differs between gut bacterial species. In conclusion, the performance of supervised classification of gut species in flow cytometry data is species-dependent, but is for some combinations accurate enough to serve as a faster alternative to 16S rRNA gene sequencing.

## Introduction

Microbial communities play an important role in global and industrial biochemical processes [1] and human health and disease [2–4]. Understanding their structure and functionality is key in manipulating microbial communities such that they carry out specific functions such as, among others, fuel production [5], plastic degradation [6], or human microbiota modulation [7].

A commonly encountered bottleneck in microbial community experiments, especially if they involve a large number of replicates, is quantifying the abundance of the different species. Conventional enumeration methods based on colony-forming units (CFU, e.g., [8]) assume that species are culturable and can be differentiated based on biochemistry or morphology. In culture-independent approaches, microbial abundance is commonly estimated by sequencing marker genes such as the 16S rRNA gene or by counting shotgun metagenomic reads mapped to genomes and reference genes [9–11]. Despite ongoing automation efforts, the entire process from DNA extraction to sequencing is still time-consuming. Varying 16S rRNA gene copy numbers, nucleic acid extraction and amplification efficiencies as well as PCR primer selectivity introduce biases that need to be corrected [12, 13]. Moreover, some form of normalization is required to adjust for different sequencing depths. The resulting relative taxon abundances do not allow accurate assessment of whether taxa change or stay constant in relation to other taxa, necessitating specific data transformation or network construction techniques to compute associations from 16S rRNA gene sequencing data [14, 15]. Additional measurements are needed to convert relative abundances to count data (e.g., quantitative PCR, quantitative sequencing spike-ins and flow cytometry [16–19]).

Flow cytometry (FC) is a single-cell technique that records optical characteristics for thousands of cells [20] and is becoming an alternative approach to sequencing for the exploration of microbial communities [21, 22]. Several tools have been developed to partly or fully automatically cluster events in FC data into groups [23–25]. In analogy to operating taxonomic units (OTUs) in sequencing data, alpha and beta diversity can be estimated based on the number of these groups [26] and differences in FC groups between samples have served as disease markers [27]. The change of FC groups over time has also been monitored to assess resistance and resilience [28] as well as neutrality [29]. However, FC groups have a disadvantage compared to OTUs: it is difficult to identify the taxa forming these groups.

Since several characteristics are measured per cell, FC data are inherently multivariate. This multivariate nature can be exploited to train a classifier on monocultures which can then be applied to assign cells in communities to different species. Thus, supervised classification techniques have the potential to deliver species-specific counts for community samples without requiring labels. In a pioneering work, neural networks applied to flow cytometry data successfully differentiated between dozens of phytoplankton species [30, 31]. This technique was also advocated by Davey & Kell for bacteria [32]. Recently, Rubbens et al. [33] were able to predict the abundances of soil bacterial species mixed in different proportions in silico as well as in vitro using linear discriminant analysis and random forests. Duygan et al. [34] applied neural networks to flow cytometry data to infer microbial ‘cell type’ diversity in a lake community by comparing samples to signatures of predefined strain and bead standards.

Human gut bacterial communities are known to be involved in gastrointestinal conditions such as inflammatory bowel disease and irritable bowel syndrome. To better understand gut microbial responses to perturbations such as antibiotics or changes in diet and to unravel microbial interactions, artificial gut communities are being studied in vitro. In such studies, species-specific microbial abundances are often assessed through 16S rRNA sequencing [35–37]. The main goal here is to evaluate the performance of supervised classification applied to FC data of human gut species and to compare it to 16S rRNA sequencing. Additionally, we look at variability of flow cytometry data across different experiments and between different gut bacteria.

## Methods

### Bacterial strains

Gut bacteria originating from human feces and a common lab strain which was also labeled with a fluorescent protein were selected for this experiment (Table 1).

**Table 1:** Characteristics of species used in this study.

Strain	Gram	Size (length – width, μm)	Shape & Occurrence	Phylum, family	Reference (Species size)	16S rRNA copy number
*Bacteroides thetaiotaomicron* DSM 2079	–	1.0–2.0 × 0.7–1.0	Oval bacillus Single or pairs	*Bacteroidetes, Bacteroidaceae*	Eggerth et al. 1932 [63]	5
*Bacteroides uniformis* RCC 1835	–	0.8 × 1.5	Bacillus Single	*Bacteroidetes, Bacteroidaceae*	Eggerth et al. 1932 [63]	4
*Bifidobacterium adolescentis* RCC 1139	+	4.0–6.0 × 1.0	Y or bifid	*Actinobacteria, Bifidobacteriaceae*	Kim et al. 2010 [64]	4
*Bilophila wadsworthia* RCC 662	–	0.7–1.1 × 1.0 -10.0	Bacillus Pleomorphic	*Proteobacteria, Desulfovibrionaceae*	Baron et al. 1989 [65]	4
*Blautia hydrogenotrophica* DSM 10507	+	0.6–0.7	Coccobacillus Pairs	*Firmicutes, Lachnospiraceae*	Bernalier et al. 1996 [56, 66]	1
*Collinsella aerofaciens* RCC 1366	+	0.3–0.7 × 1.2 - 4.3	Cocci Chain of 6-120 cells	*Actinobacteria, Coriobacteriaceae*	Kageyama et al. 1999 [67]	5
Escherichia coli* DSM 6050	-	1.0–2.0 × 0.5	Bacillus Single	*Proteobacteria, Enterobacteriaceae*	Riley, 1999 [68]	7
*Faecalibacterium prausnitzii* DSM 17677	–	0.5–0.8 × 2.0–9.0	Bacillus	*Firmicutes, Clostridiales*	Duncan et al. 2002 [69]	6
*Parabacteroides merdae* RCC 643	–	0.8–1.6 × 1.2–12	Bacillus Single	*Bacteroidetes, Porphyromonadaceae*	Sakamoto et al. 2006 [70]	7
*Prevotella copri* DSM 18205	–	0.83 × 0.91–0.99	Bacillus Single	*Bacteroidetes, Prevotellaceae*	Hayashi et al. 2007 [71]	4
*Roseburia intestinalis* DSM 14610	Gram variable(+)	0.5 × 1.5–3.0	Bacillus	*Firmicutes, Lachnospiraceae*	Duncan et al. 2002 [72]	6
*Ruminococcus bromii* RCC 1377	+	0.7–1.1(diameter)	Cocci Pairs and chains up to 8 cells	*Firmicutes, Ruminococaceae*	Moore et al. 1972 [73]	4

DSMZ: German Collection of Microorganisms and Cell Cultures GmbH. RCC: Raes lab Culture Collection. Cell size and shape are reported for different media from literature. Cell size is known to be variable depending on growth phase and medium used. 16S rRNA copy numbers were taken from rrnDB [44]. Bacillus shape: rod-like. Coccus shape: Sphere-like.

* With and without plasmid pRSetB-mCherry.

### Culture conditions

All bacteria were cultivated at 37 °C without agitation under anaerobic conditions in a Don Whitley A135 Anaerobic Workstation with HEPA filter (10% H_2_, 10% CO_2_, 80% N_2_, 55% humidity). 16S rRNA gene sequencing was performed regularly to confirm species identity.

Prior to the experiments, all gut-derived strains were subcultured twice (48 h and 18 h, respectively) in modified Gifu Anaerobic Medium broth (mGAM [38], HyServe), except for *F. prausnitzii* DSM17677, which was grown in Reinforced Clostridial Medium broth (RCM, Oxoid). All bacterial cultures were sampled in stationary phase, as determined by optical density OD_600_ in a plate reader (Epoch2, Biotek, Supplementary Fig. 1).

*E. coli* expressing mCherry was cultivated at 37 °C in RCM broth, in the presence of ampicillin (100 μg/ml), with 200 rpm agitation and under aerobic conditions for 16 hours prior to each experiment.

### Flow cytometry

After 18 hours of growth, the cells were serially diluted 1000× in PBS to an approximate cell density of 10^6^ cells per ml and stained with 1 μl/ml SYBR green I (1:100 dilution in dimethylsulfoxide; 20 min incubation at 37 °C; 10.000 concentrate, Thermo Fisher Scientific) following the protocols described in [18, 33, 34, 39]. Two flow cytometers were used in this study. The setup and experimental use of both instruments are summarized in Table 2. For selected mock communities, we used a benchtop Accuri C6 flow cytometer (BD Biosciences). A threshold value of 2000 was applied to the FL1 channel. The Accuri C6 flow cytometer delivered a multiparametric description of each event in each  sample consisting of 13 parameters (SSC-A, SSC-H, FSC-A, FSC-H, FL1-A, FL1-H, FL2-A, FL2-H, FL3-A, FL3-H, FL4-A, FL4-H and Width). During the study period, the instrument was calibrated daily with Spherotech 8-peak and 6-peak validation beads (BD Biosciences). The four-species community as well as all mock communities involving *E. coli* with mCherry were measured with a benchtop CytoFLEX S flow cytometer (Beckman Coulter) which, in contrast to Accuri C6 instrument, has the required filters to detect mCherry (mainly 610/20). This resulted in a multiparametric description of each event consisting of 23 parameters (FSC-A, FSC-H, SSC-A, SSC-H, FL1-A, FL1-H, FL2-orange-A, FL2-orange-H, FL3-red-A, FL3-red-H, FL4-A, FL4-H, APC-A750-A, APC-A750H, VSSC-A, VSSC-H, KO525-A, KO252-H, mCherry-A, mCherry-H, PI-A, PI-H and FSC-Width). During the study period, the instrument was calibrated daily with CytoFLEX Daily QC Fluorospheres.

**Table 2:** Flow cytometers used in this study

Flow cytometer	Laser	Detector	Filter	Used in figure
Accuri C6	Blue (488 nm)	FL1	533/30	2, 4 A, 6
		FL2	585/40	
		FL3	670 LP	
	Red (640 nm)	FL4	675/25	
CytoFLEX S	Violet (405 nm)	VSSC	405/10	3, 4CDE, 5
		KO525	425/40	
	Blue (488 nm)	FL1/GFP	525/40	
		FL4	690/50	
	Yellow (561 nm)	FL2	585/42	
		mCherry	610/20	
		PI	690/50	
	Red (638 nm)	FL3	660/10	
		APC-A750	780/60	

All events were quantified using a volumetric method (measured events/μl).

### In vitro mock communities

**Fig. 1:**
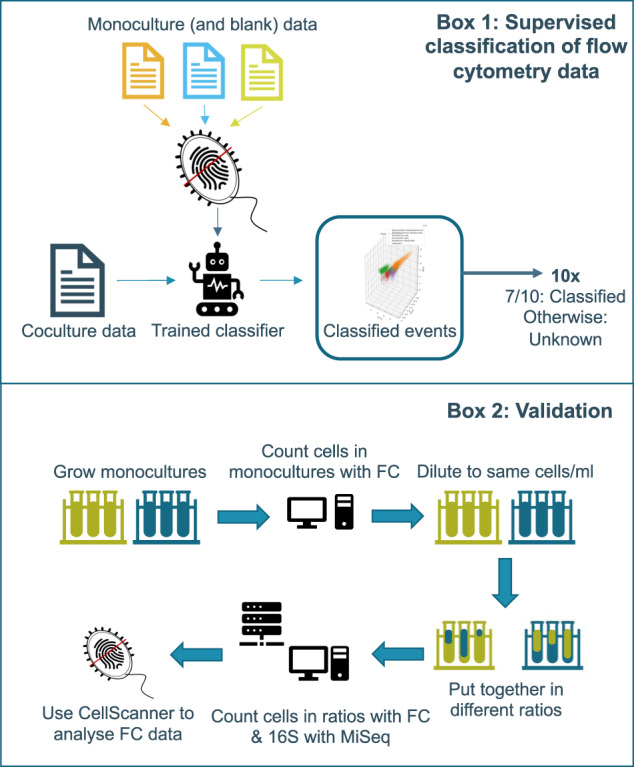
A brief overview of CellScanner is shown in box 1. A classifier is trained on monocultures and blank data and subsequently applied to coculture data to classify every event ten times. When the repeated classifications agree on one species (or blank) for >70%, the event will be classified as such. Otherwise, the event will be classified as “unknown”. Box 2 summarizes the collection of mock community data. In short, monocultures were grown to stationary phase, cells were counted with a flow cytometer, and then diluted to obtain an equal number of cells per ml. These dilutions were used to make different ratios of the bacteria. Subsequently, mock communities were subjected to flow cytometry (FC) and 16S rRNA gene sequencing with Illumina MiSeq. Finally, FC data were analyzed with CellScanner.

Cell densities of all in vitro mock communities were first measured separately with the CytoFLEX, after which the community suspensions were diluted in 0.2 μm filtered PBS to reach final cell densities of approximately 5000 cells/μl. These standardized suspensions were then mixed in intended proportions of 5%, 10%, 20%, 40%, 50%, 60%, 80%, 90 and 95%, in a final volume of 1 ml (Fig. 1). In the case of the mock community with *Collinsella aerofaciens* and *Bacteroides thetaiotaomicron*, proportions of 1 and 2% were also prepared. Due to inherent pipetting errors, the mock communities do not reach the intended proportions precisely. For this reason, cell density in each monoculture (for the intended proportions) was counted with the flow cytometer by diluting the cells in 0.2 μm filtered PBS. The proportions based on these measurements differ somewhat from the intended proportions and are referred to here as expected proportions. Proportions predicted through supervised classification are compared to these expected proportions. Removal of debris and/or background was accomplished here by gating for SYBR/mCherry events in FL1 and FL3 channels respectively.

### In vitro co-growth communities

The in vitro co-growth community was composed of four bacteria (*Roseburia intestinalis, Blautia hydrogenotrophica, Bacteroides thetaiotaomicron* and *Faecalibacterium prausnitzii*). Monocultures were grown for 18 hours in RCM, after which cells were counted with the CytoFLEX flow cytometer and cultures were diluted to roughly obtain 1*10^6^ cells per ml per species. Next, bacteria were added in equal proportions to obtain a total final concentration of 4*10^6^ cells/ml in 10 mL RCM. Both monocultures and communities were grown for 48 hours in batch, and samples were taken at timepoints 24 h and 48 h. Three biological replicates were prepared in this experiment.

### 16S rRNA gene sequencing

Samples were centrifuged at 12130 × *g* for 10 min. The supernatant was removed, and the remaining pellets were stored at −80 °C until further processing. DNA extraction was carried out using the MoBio PowerMicrobiome RNA isolation kit as previously described [40]. Next, the V4 region for the 16S rRNA gene was amplified with the primer pair 515 F/806 R and sequencing was performed using the Illumina MiSeq platform to generate paired-end reads of 250 base pairs. After demultiplexing with sdm as part of the LotuS pipeline [41] without allowing for mismatches, fastq sequences were preprocessed using DADA2 pipeline v1.14.1 [42]. The taxonomy was assigned initially using RDP classifier v2.13 but for taxa that were not correctly identified, the sequence variants were aligned to EzBioCloud database [43] to ensure accurate assignment of the species. Taxa proportions were corrected with 16S rRNA gene copy numbers retrieved from the rRNA operon copy number database rrnDB [44] and the National Center for Biotechnology Information (NCBI, Bethesda (MD): National Library of Medicine (US)), and multiplied by total cell count from the sample obtained by flow cytometry.

### CellScanner

CellScanner is a new standalone tool (manuscript in preparation) for the analysis of flow cytometry data that performs gating and uses supervised classification techniques to assign events from cocultures to species indicated by the user (Fig. 1). CellScanner relies on flow cytometry data from monocultures (reference files) to train 10 classifiers (neural networks of 200 layers, using lbfgs solver and the rectified linear unit activation function). For each classifier, 1000 events per species are selected randomly from the corresponding monoculture. Of these, 875 events (7/8) are used to train the neural network and 125 events (1/8) to test the trained neural network. Each trained classifier then assigns a species to each event in the coculture. This procedure is carried out for each co-culture sample separately. For the analyses on Accuri data, all 13 parameters were taken into account. For CytoFLEX, nine parameters representing the area (FSC-A, SSC-A, FL1-A, FL2-orange-A, FL3-red-A, FL4-A, VSSC-A, mCherry-A, PI-A) and FCS-Width were considered. Only the area (A) records were considered since the height (H) records did not provide additional information and increased the calculation time.

For each experiment, monoculture data from the same experiment was used to train the model.

If the mCherry protein is excited, the emission will be detected by the following filters: 660/10, 690/50, 780/60, 585/42, 610/20 and 690/50. For the classification of *E. coli* and *R. intestinalis* without taking the red-fluorescence of mCherry into account, only the following parameters were used in CellScanner: FSC-H, FSC-A, SSC-H, SSC-A, FL1-H, FL1-A, VSSC-H, VSSC-A, KO525-H, KO525-A and FSC-Width, thus removing the information from the filters detecting mCherry.

For data obtained by the Accuri flow cytometer, events were identified as background if they met the criteria given by at least one of the following equations, matching the gating described by Vandeputte et al. [18]:

$$FL3A = = 0\,or\,FL1A = = 0$$

$$FL3A \; > \; 0,0241 \times FL1A^{1.0996}$$

$$FSCA \; > \; 100000\,\& \,SSCA \; > \; 10000$$

Because of the stringent threshold settings of the Accuri, very few blank events were detected (<100 per sample). This “line gating” method was thus sufficient to limit the effect of background events on the prediction. For data obtained from CytoFLEX, the considerable amount of detected debris was removed by supervised classification (machine learning). Ten classifiers were trained on blanks and samples from a monoculture respectively to differentiate between debris and cells from a single species. The events classified as debris or as “unknown” were removed. This machine-learning based gating was repeated for each monoculture.

CellScanner was applied to community files that originated either from cocultures in vitro or were compiled in silico from monoculture files (5000 maximum events per species). In the latter case, 1000 events were selected from each monoculture file. Since 10 classifiers are trained, 10 species assignments are made for each event in the coculture. When the ‘unknown’ setting is enabled, and seven out of the ten classifiers agree on a species, then the event is assigned to the corresponding species, else it is classified as unknown. Without the ‘unknown’ setting, each event is classified according to the majority vote of the classifiers. In case of a tie, the event is randomly classified as one of the species.

CellScanner calculates the accuracy (ACC) and the F1 score:

$$Accuracy = \frac{{TP + TN}}{{P + N}}\,F1\,score = \frac{{2TP}}{{2TP + FP + FN}}$$

With TP= True positive, P =Positive values, N = Negatives values, TN = True negative value, FP= False positive value and FN= False negative values.

The removal of events classified as ‘unknown’ reduces the number of false positives, which increases the specificity (TN/(TN+FP)) and the precision (TP/(TP+FP)) (Supplementary Fig. 2). Unless stated otherwise, the “unknown” setting was enabled and proportions were calculated after removal of unknown events.

For ease of comparison, relative abundances are shown. The absolute abundances as events measured by flow cytometry and classified by CellScanner are reported in Supplementary Tables 1–6.

### In silico communities

All in silico communities consisted of monoculture data from cells grown in mGAM derived from separate FC measurements performed on the Accuri C6 flow cytometer. The in silico communities were generated by CellScanner, using a maximum of 1000 random events from each monoculture file to create a community file.

### Feature importance

LIME (Local Interpretable Model-agnostic Explanation, [45]) was applied to assess the importance of different flow cytometer parameters (= features) for species classification in 66 pairwise species combinations. The importance of each of the 13 Accuri features was estimated for 50 events per classifier, running 10 classifiers per combination (i.e., 500 events). The more predictive a feature is for either species, the higher is the importance of that feature. Importance values receive a positive or negative sign depending on whether the feature contributes to classifying an event as belonging to a species or as not belonging to it. We took the absolute of each importance value and summed feature values coming from the same detector (A+H). We calculated the mean importance for the seven parameters (FSC, SSC, FL1, FL2, FL3, FL4 and Width) for each pair across the 500 events and subsequently for each shape combination.

### Intraspecies variation

To assess intraspecies variation, we analyzed monocultures of four different species (*Escherichia coli, Bacteroides thetaiotaomicron, Blautia hydrogenotrophica, Roseburia intestinalis*) and the medium alone (mGAM), using seven monocultures from different dates for each. A thousand events from each experiment were selected randomly, except for blank controls, for which all events were considered (0-70 events per file). We ran CellScanner on the five-species in silico community (four species + blank) with the majority rule described above (events on which less than 70% of classifiers agreed were assigned as “unknown”). We calculated pairwise Euclidean distances between events from the Accuri flow cytometer with R function daisy in the cluster package. Because the FL4 parameter is not predictive and highly variable within every monoculture on Accuri, we removed it from the distance calculation to avoid artefacts.

### Statistical analysis

All statistical analyses were performed using R (version 3.6.1, http://www.R-project.org).

## Results

### Identification of gut bacterial species in in silico communities

**Fig. 2:**
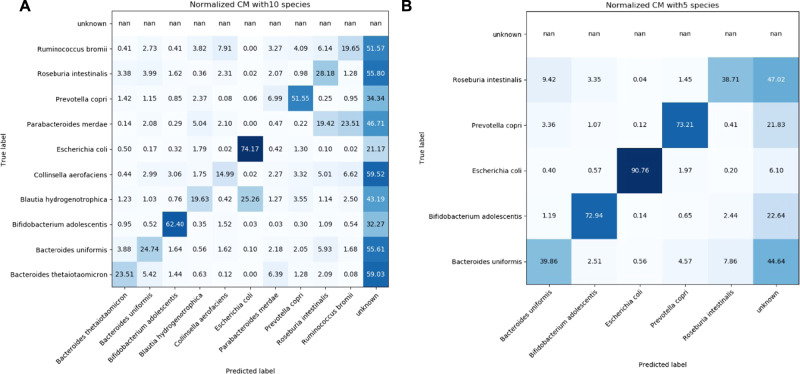
Accuracy of species identification in FC data of gut bacterial communities compiled in silico. Confusion matrices for an in silico 10-species community (**A**) and 5-species community derived from the five best-identifiable species from A (**B**). Events in monoculture data obtained with Accuri C6 were gated and mixed in equal proportions in silico. The true species is depicted on the y-axis, the predicted species is depicted on the x-axis. The numbers are the percentages of the events that are classified as such. The higher the number in the diagonal, the more accurately CellScanner predicted the species.

We first tested how accurately gut bacterial cells can be identified in an in silico mixture, where the true positive and false negative assignment for each event in the community is known. For this, we collected flow cytometry data of monocultures for ten gut species in stationary phase with Accuri C6, mixed them in silico in equal proportions and quantified the accuracy of species identification in these mixtures with CellScanner (Fig. 2A).

For half of the species, more than 50% of the events were not consistently assigned to a single species and thus classified as “unknown”. This suggests that many species overlap in the measured features, which makes them difficult to distinguish by machine learning. Some species, such as *Escherichia coli*, have distinct features and are thus easy to classify (Supplementary Fig. 3), while the features of other species, e.g., *Bl. hydrogenotrophica*, overlap with another species, resulting in misclassification. Of note, two species belonging to the same genus (*Bact. thetaiotaomicron* and *Bact. uniformis*) are not misclassified as each other but are more commonly misclassified as species in other genera. The overall accuracy of species identification in this ten-species community is 32%, with an F1 score of 39% (Supplementary Fig. 3), including the events assigned as “unknown”. When reducing the community to five species (Fig. 2B), the overall accuracy almost doubles to 62% and the F1 is 71% (Supplementary Fig. 4). With less overlap between the species, the individual classification true positive rate reaches a minimum of 39% for all species, and the proportion classified as ‘unknown’ decreases substantially.

As expected, when selecting the five species that were easiest to recognize in the ten-species community the accuracy of species identification increased. This could help in species selection when designing a consortium.

### Quantification of species in vitro with mock communities

**Fig. 3:**
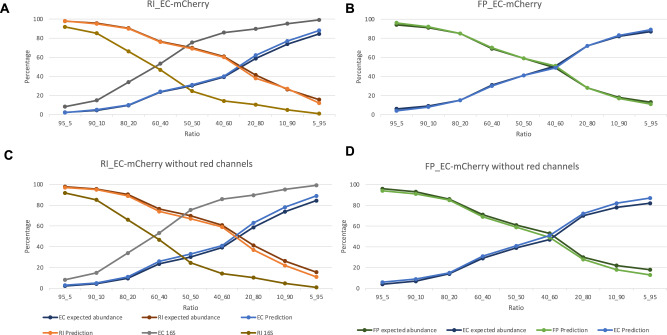
Comparison of supervised classification of flow cytometry data and 16S rRNA gene sequencing on mock communities. **A** Expected proportions and proportions predicted with CellScanner for *R. intestinalis* (RI) and *E. coli* (EC), the latter expressing mCherry. 16S rRNA gene sequencing results are included. **B** Expected proportions and proportions predicted with CellScanner of FP (*F. prausnitzii*) and EC, the latter expressing mCherry. **C** Expected proportions and proportions predicted with CellScanner, without using the red channels in the classification, of RI and EC expressing mCherry. 16S rRNA gene sequencing data are included. **D** Expected proportions and proportions predicted with CellScanner, without using the red channels in the classification, of FP and EC expressing mCherry. All flow cytometry shown here were obtained with CytoFLEX.

To test whether we can accurately quantify species in a mixture in vitro, we mixed three gut bacterial species grown to stationary phase (*E. coli* expressing mCherry, *R. intestinalis* and *F. prausnitzii*) in different proportions, resulting in three combinations of two species. Labeled *E. coli* was included as a positive control because the CytoFLEX flow cytometer can easily distinguish the mCherry colored *E. coli* cells from the SYBR Green stained *R. intestinalis* or *F. prausnitzii* cells (Fig. 3). The proportions predicted with CellScanner and with 16S rRNA gene sequencing were compared to the expected proportions. As shown in Fig. 3A and C, the prediction of CellScanner is almost identical to the expected proportions, with an absolute mean difference of 1%. The absolute mean difference of the 16S results is 25% from the expected proportions, with 2% of the number of events classified as ‘unknown’ on average. For ease of comparison, proportions are compared, absolute abundances for all species combinations are reported in supplement (Supplementary Table 1).

Because it is straightforward to distinguish *E. coli* labeled with mCherry from a non-labeled species, we tested whether CellScanner could still differentiate the two species when the red fluorescence channels were left out in the software, in essence removing the fluorescent label of *E. coli*. As shown in Figs. 3C and 3D, CellScanner predictions are less accurate without these channels, with events classified as ‘unknown’ increasing to 8% (Supplementary Table 2) but are still close to the expected abundances (absolute mean abundance difference of 3%). Thus, for these species pairs, information from scattered light and the remaining channels was sufficient to accurately identify both species in the mixture.

**Fig. 4:**
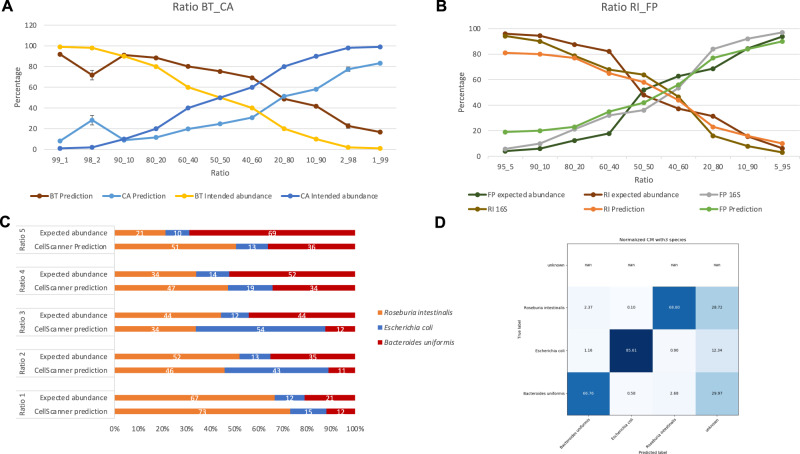
Proportions in two- and three-species mock communities predicted through supervised classification of flow cytometry data. **A** Intended and predicted proportions for *Bact. thetaiotaomicron* (BT) and *C. aerofaciens* (CA). In contrast to expected proportions, intended proportions have not been corrected for pipetting errors (see Methods). Error bars: Standard deviation over two technical replicates. Flow cytometry was carried out with Accuri C6. **B** Expected proportions and proportions predicted by CellScanner for *R. intestinalis* (RI) and *F. prausnitzii* (FP). 16S rRNA gene sequencing data are included. **C** Proportions predicted by CellScanner for a three-species mock community of *R. intestinalis, E. coli* and *Bact. uniformis*. **D** Confusion matrix of an in silico three-species community made from the monocultures of the species in (**C**). For panels (**B**–**D**), flow cytometry was carried out with CytoFLEX.

Next, we tested how well CellScanner could identify unlabeled species in stationary phase in mock communities of known proportions. First, we collected mock community data for 11 ratios of *Bact. thetaiotaomicron* and *C. aerofaciens* and found that proportions predicted by CellScanner are relatively close to the expectation, with an absolute mean difference of 19% (Fig. 4A, Supplementary Table 3). We then repeated this experiment for *F. prausnitzii* and *R. intestinalis*. For comparison, we also determined mock community proportions through 16S rRNA gene sequencing. In case of *F. prausnitzii* and *R. intestinalis*, both sequencing and CellScanner are close to the expected abundance (absolute mean difference of 7% for 16S versus 13 and 18% for *F. prausnitzii* and *R. intestinalis* respectively; Fig. 4B and Supplementary Table 4).

For a mock community of three species, the absolute mean difference between the expected abundance and CellScanner’s prediction is 13%, 17 and 23% for *R. intestinalis, E. coli and Bact. uniformis* respectively (Fig. 4C; Supplementary Table 5 shows the results when keeping ‘unknown’ events). Although the confusion matrix (Fig. 4D) shows that *E. coli* should be easily distinguished from the other bacteria, it is not always predicted in the correct proportion (e.g., Fig. 4C, Ratio 3). In conclusion, supervised classification works well for some bacterial species combinations but not for others, in agreement with previous findings [33].

**Fig. 5:**
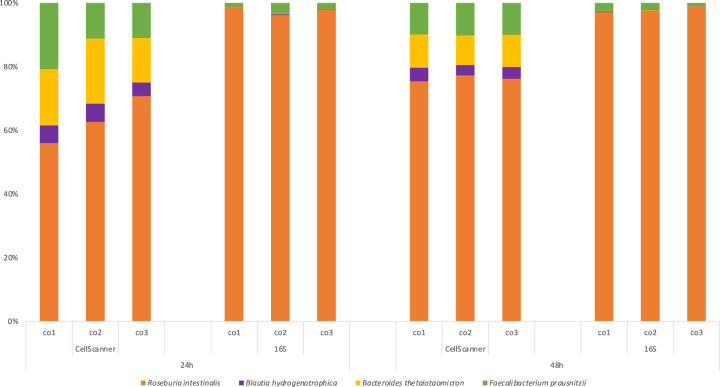
Comparison of 16S rRNA gene sequencing and supervised classification of flow cytometry data for a four-species coculture grown for 48 hours. In this case, the ‘unknown’ setting (see Methods) was switched off, since more than half of the events was classified as ‘unknown’ (RI dominance is also seen when keeping unknowns, see Supplementary Fig. 5). Three biological replicates are shown as co1, co2, and co3. Flow cytometry was carried out with CytoFLEX.

The experiments described above were performed with in vitro mock communities with abundance measurements available for each species for each ratio. Finally, we tested whether CellScanner could identify species in a community of four gut bacteria grown together for 48 h. Since 50% of the events were classified as ‘unknown’ using the settings as described in the “Methods” (Supplementary Fig. 5 and Supplementary Table 6), we ran CellScanner without any “unknown” assignment to assess how these previous “unknown” events were classified (Fig. 5). In both cases, CellScanner and 16S rRNA gene sequencing agree on *R. intestinalis* dominance after 24 and 48 h of growth. Although CellScanner’s accuracy drops with increasing species number, prediction of bacterial dominance is still in agreement with sequencing results in this case.

### Shape and size differences do not explain classification accuracy

**Fig. 6:**
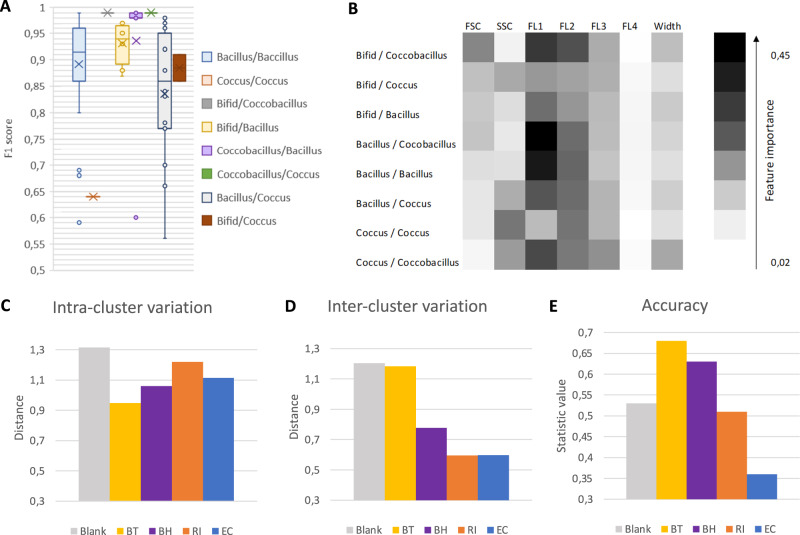
Impact of shape, features and heterogeneity on classification accuracy. **A** F1 score for pairwise in silico predictions within 12 species (66 combinations) grouped by shape (i.e., Bacillus, coccus, bifid, coccobacillus). **B** Feature importance calculated with Lime. **C**–**E** Heterogeneity assessed for seven biological replicates, for four species, *Bact. thetaiotaomicron* (BT), *Bl. hydrogenotrophica* (BH*), R. intestinalis* (RI), *E. coli* (EC), and medium debris (Blank). **C** Intra-cluster variation is computed as the mean pairwise Euclidean distance averaged across experiments per species. **D** Inter-cluster variation is assessed as the mean of all pairwise centroid distances per species, where a centroid is computed for each species-specific experiment. Accuracy refers to CellScanner accuracy when assigning events from merged replicates to the correct experiment of origin. A low intra-cluster variation combined with a high inter-cluster variation reduces the overlap between experiment-specific clusters and results in a high accuracy.

Flow cytometry data depends on physical characteristics of a cell such as size (forward scatter) and shape (side scatter). To test whether differences in size and shape improve classification accuracy, we compared all 66 pairwise predictions for 12 gut bacterial species in silico (Supplementary Fig. 6) and found that difference in shape is not sufficient to ensure a high accuracy (>80%, Fig. 6A) and that vice versa, species pairs with the same shape can reach high accuracies. For instance, 89% of the pairwise predictions within the bacillus group resulted in an F1 score greater than 80%. Next, we compared the importance of different features of FC data (i.e., forward scatter, side scatter etc.) for species classification. The feature importance was calculated with Lime (see “Methods”), a program that compares the feature value for each event to the feature values of the training dataset with similar values to assess with which probability this feature represents a specific species. This way, Lime assesses whether a particular feature makes a useful contribution to the classification task.

We assessed feature importance for all 66 pair-wise predictions for 12 bacteria in silico (Fig. 6B) and confirmed that neither size nor shape was sufficient to separate species. For all predictions, forward and side scatter had a lower importance (respectively 10 and 12% of the global importance on average) than the other features together (78% of the global importance on average), but they also contributed to classification. Thus, more than two features are needed to separate the species with high accuracy, emphasizing that multivariate data are necessary for classification. As expected, the feature importance of fluorescence channels decreased with their distance to the SYBR-Green emission spectrum, with FL-1 having the highest and FL-4 the lowest values. In addition, we found that 50 of the pairwise predictions with a forward scatter feature importance value higher than 20% were related to *Bif. adolescentis* (bifid group). This suggests that the shape of this species is distinct enough from the others to have an impact on species classification.

### Species properties in flow cytometry data differ across biological replicates

To assess the robustness of the predictions to biological variation, we tested whether CellScanner could distinguish monoculture data of the same species across biological replicates. We mixed monoculture data of each species to obtain in silico communities*. Bact. thetaiotaomicron* biological replicates are separated with an accuracy of 68% and *Bl. hydrogenotrophica* with 65%, for seven monocultures each. The other species are harder to distinguish between experiments, in particular *E. coli* for which we observed an accuracy of 36% (Fig. 6C). Differences between monocultures of *Bact. thetaiotaomicron* are already apparent in the 3D plot, where the clusters are distinct (Supplementary Fig. 7).

To confirm that monocultures of the same species can vary from one experiment to another, and to emphasize that this variation depends on the species, we assessed intra- and inter-cluster variation (Fig. 6C). We computed intra-cluster variation as the mean of all pairwise Euclidean distances between events per experiment and then averaged across all experiments of a species. For inter-cluster variation, we computed the mean pairwise Euclidean distance between experiment centroids, where the centroid is defined as the mean distance between experiment-specific events. A large intra-cluster variation is due to high heterogeneity within experiments whereas a large inter-cluster variation indicates high variation between experiments. We also assessed the variability across technical replicates, which is small compared to biological replicates (Supplementary Fig. 8, Supplementary Table 3). These results confirm that clusters change across biological replicates, and that this change is species-specific (e.g., strong for *Bact. thetaiotaomicron* and weak for *E. coli*).

We observed the highest intra- and inter-cluster variation for the blanks (Fig. 6C), which we attribute to the small number of particles and to their high diversity. Because the size and shape of particles in the blanks differ, they do not always form a well-defined cluster. However, their features are distinct enough to separate these particles from events representing bacterial cells using supervised classification.

In summary, heterogeneity across biological replicates is variable and species-specific.

## Discussion

In this study, we evaluated supervised classification applied to FC data as a method to count gut bacterial species in mixtures. Assessment of this method on mock communities in silico and in vitro showed that it can resolve proportions in cocultures, but also that its accuracy depends on the species combination. In addition, in a low-complexity gut community, it reproduced trends seen with 16S rRNA gene sequencing.

Our method has several advantages: it avoids labor-intensive DNA extraction or plating, does not require fluorescent labeling of species, and in contrast to 16S rRNA gene sequencing delivers absolute abundances. However, the method is limited to cocultures and small bacterial communities; we observed a decline in accuracy with increasing species number (Fig. 2).

In the co-growth experiment (Fig. 5), it is of note that *Bl. hydrogenotrophica* disappears from the second replicate in 16S data, but not in FC-based data. It may still be present in 16S rRNA gene sequencing data but was too rare to be captured during sequencing. Alternatively, in FC data, cells from other species may have been misclassified as *Bl. hydrogenotrophica*, inflating its abundance in FC-based counts (Supplementary Fig. 9). However, 16S rRNA gene sequencing accuracy in small communities can also be low. For example, the 16S rRNA gene sequencing results differed on average 25% for the expected abundance in the mock community with *E. coli* expressing mCherry (Gram-negative) and *R. intestinalis (*Gram-positive), but only 9% in the community with *R. intestinalis* and *F. prausnitzii*, where both bacteria are Gram-positive. This variation is in line with previous studies showing that 16S rRNA gene sequencing results of mock communities did not match the expected community compositions [46–48]. In the absence of a ground truth for the co-growth experiment, we do not know which technique is closer to the true counts.

We show that FC features linked to cell shape and size are not sufficient to distinguish species. FC-based data are commonly analyzed by incorporating one or two features at the same time (2-D histograms). In the present study, feature importance was often evenly spread across three or more features when classifying species pairs and also included spillover channels. This is in line with previous experiments [49], where the authors show (using the same FC instrument), that FL1, FL2, FSC and FL3 are the channels resulting in the best identification. In addition, they note that with increasing community complexity, more channels are needed for an optimal identification. It is therefore important to use multivariate methods to benefit from all generated data in order to classify each event with higher accuracy. In our experiments, a single non-discriminating dye (SYBR Green) staining all cells was combined with an *E. coli* strain expressing a fluorescent protein to be able to distinguish it from other species. As expected, a species-specific fluorescent label increased the accuracy of supervised classification (Supplementary Table 7). Likely, further combinations with other fluorescent labels allowing to distinguish different species may lead to an increased accuracy of single-cell predictions in (complex) microbial communities [50, 51]. For instance, a fluorescent polyclonal antibody against *F. prausnitzii* was developed recently for use in flow cytometry [52].

For some species such as *E. coli*, variability across biological replicates of monocultures was low, while other bacteria (e.g., *Bact. thetaiotaomicron* and *Bl. hydrogenotrophica*) showed considerable variation. We found technical variability to be consistently small (Supplementary Fig. 8), implying that the variability mostly had biological sources. Vives-Rego et al. [53] hypothesized that both cell size diversity and cell cycle variations lie at the origin of experimental variation. This can influence the monoculture data when comparing datasets from different experiments with the same bacterial monoculture measurements. We tried to keep this to a minimum in our experiments by using bacteria in the stationary phase of the growth cycle and using the same medium for each experiment. Another reason for the variability could be explained by bacterial aggregation, which may differ for each species per experiment and could influence the measured parameters [54]. We accounted for this by vortexing the samples, but since we are not sure whether this resolved the issue, it could be further explored in future studies. In the case of *Bact. thetaiotaomicron*, biological variability can be attributed to cell shape switching between three morphologies resembling the Greek letters θ, ι and ο [55]. However, cell shape variation is probably not the only factor explaining biological variability since heterogeneity is also observed for other bacteria with only one cell shape such as *Bl. hydrogenotrophica* [56]. The latter species can occur singly or in pairs, which might affect the readings in the flow cytometer if they cross the light beam when still attached to each other. An important additional limitation of our method is the observation that coculturing bacteria can lead to reduced phenotypic heterogeneities [57], and therefore the characteristics measured in monocultures might not always represent the same characteristics when grown in coculture. Future research could identify the traits that affect the features measured by FC. If one of the species in the coculture could be distinctly labeled, classifiers could be trained on these events without taking the channels used for the label into account. Subsequently, these classifiers could be used on a community without labeled species. Although this would require initial labeling of species, the labeling step(s) could be omitted later allowing for a more efficient throughput of samples. The need for training data (and hence monocultures) for different media and physiological states could be overcome with unsupervised clustering approaches. However, such approaches would require a method of linking clusters to species and may not be sufficiently accurate. Alternatively, a publicly available collection of monocultures and trained classifiers built by the research community could address this problem in the long term.

The method was tested on gut bacteria in stationary phase. Since cells may change their physiology throughout the growth curve, it is a limitation of this method that mono- and coculture samples need to be taken from the same growth phase. In addition to using calibration beads to calibrate the flow cytometer, a standardized bacterial mock community could be used to account for differences in sample handling during different FC experiments [58].

The field of flow cytometry is constantly evolving and detection of small particles is getting more accurate. As the resolution of FC instruments increases, we are able to obtain more detailed data for each event, which will increase classification accuracy. In addition, better and smaller cameras improve imaging technologies for FC (IFC), which allows capturing a photo of each individual event that could subsequently be automatically analyzed. IFC can capture multiple cellular parameters such as size, volume, and shape [59–61]. Combining the multiparametric data from conventional FC and IFC could further boost the accuracy of supervised classification. In addition, more recent machine learning techniques, such as UMAP, may outperform the classification technique used here [62].

Taken together, our results illustrate that machine learning combined with FC can give accurate abundances for unlabeled species in cocultures and captures trends in small communities. In combination with multiplex labeling, this approach has the potential to become a fast yet accurate technique for differential counting of microorganisms in small communities.

## Supplementary information

Supplementary Figures

Supplementary Table 1

Supplementary Table 2

Supplementary Table 3

Supplementary Table 4

Supplementary Table 5

Supplementary Table 6

Supplementary Table 7

## Data Availability

CellScanner is available on GitHub: https://github.com/Clem-Jos/CellScanner. All flow cytometry data is available on flowrepository.org. To open the link, please paste it into a browser. Ratios BT & CA: https://flowrepository.org/id/FR-FCM-Z3TX Ratios RI, FP & EC: https://flowrepository.org/id/FR-FCM-Z3TM Ratios RI, BU & EC https://flowrepository.org/id/FR-FCM-Z3TP Cogrowth of RI, BH, BT & FP: https://flowrepository.org/id/FR-FCM-Z3TQ Monoculture data: https://flowrepository.org/id/FR-FCM-Z3U2
