# Supplementary material for: Fast quantification of gut bacterial species in cocultures using flow cytometry and supervised classification
Source: ISME Commun. 2022 Apr 25;2:40. doi: 10.1038/s43705-022-00123-6 (PMC9723706; doi:10.1038/s43705-022-00123-6)
Supplement: Supplementary file 1 — Supplementary Figures [file 43705_2022_123_MOESM1_ESM.docx]

**Supplementary Figures:** **Fast quantification of gut bacterial species in cocultures using flow cytometry and supervised classification**

**Figure S1**: Growth curves of *Roseburia intestinalis* (RI), *Blautia hydrogenotrophica* (BH), *Bacteroides thetaiotaomicron* (BT), *Faecalibacterium prausnitzii* (FP), *Prevotella copri* (PC), *Escherichia coli* (EC), *Collinsella aerofaciens* (CA) and *Bacteroides uniformis* (BU). Optical density OD600 measured for 18 hours with the Epoch2 (Biotek) platereader. Error bars represent the standard deviation across three replicates. No replicates available for BU.

**Figure S2**: Effect of the assignment of unknown events on statistical measures (specificity, sensitivity, precision, accuracy and F1 score). Events are classified as unknown when 70% or fewer classifiers agree on the classification.

**Figure S3**: 3D plots of an in silico community with 10 species. A: Expected results. B: Predicted results, including unknown assignment. Data obtained by the Accuri C6 flow cytometer.

**Figure S4**: 3D plots of an in silico community with five species. A: Expected results. B: Predicted results, including unknown assignment. Data obtained by the Accuri C6 flow cytometer.

**Figure S5**: CellScanner prediction with unknown, renormalized prediction without unknown and 16S rRNA gene sequencing results for a four-species community with three replicates. Samples were taken 24 and 48 hours after inoculation. Renormalization refers to the calculation of percentages after removal of unknown events.

**Figure S6**: Accuracy and F1-score table for the 64 in silico cocultures for all pairwise combinations of 12 gut bacterial species.

**Figure S7**: Intraspecies variation analysis for seven monocultures for in silico communities. A: Graphic representation of predicted and expected values for a seven-monoculture in silico community of *Bacteroides thetaiotaomicron* (BT) and B: *Escherichia coli* (EC). C: Confusion matrix (CM) comparing predicted result to expected result for the in silico community of BT and D: EC*.*

**Figure S8**: Intra-species variation is low in technical replicates: CellScanner was trained with two technical replicates as two different species and produced a prediction on an in silico community with the same merged information. An accuracy value is calculated per prediction. Each species is tested with a set of four technical replicates. CellScanner is used with machine gating and the unknown setting activated. The random results are predictions where each classifier is replaced by randomly assigning events to the two technical replicates and calculating a mean prediction with the unknown setting activated. The accuracy of all random predictions is lower than 10% with the majority of the events classified as unknown. The accuracy for all predictions is lower than 50%, on average 22%. More than 50% of the cells are classified as unknown. Thus, supervised classification is not able to differentiate well between technical replicates.

**Figure S9**: Confusion matrices of training data for the co-grown community after (A) 24 and (B) 48 hours. The true species is depicted on the y-axis, the predicted species is depicted on the x-axis. The numbers are the percentages of the events that are classified as such. The higher the number in the diagonal, the more accurately CellScanner predicted the species.
